# 1,3,5-Tris{[3-(1*H*-benzotriazol-1-ylmeth­yl)phen­oxy]meth­yl}-2,4,6-trimethyl­benzene

**DOI:** 10.1107/S1600536808028845

**Published:** 2008-09-13

**Authors:** Chen Xu, Wan-Ling Si, Zhi-Qiang Wang, Hong-Ji Ma, Bao-Ming Ji

**Affiliations:** a College of Chemistry and Chemical Engineering, Luoyang Normal University, Luoyang 471022, People’s Republic of China; b Department of Chemistry, Henan Institute of Education, Zhengzhou 450014, People’s Republic of China

## Abstract

In the title compound, C_51_H_45_N_9_O_3_, three 1-(1*H*-benzotriazol-1-ylmeth­yl)-3-phen­yloxy (bmph) ligands are bonded to the central benzene ring in an asymmetric arrangement, two bmph located on one side of the central benzene ring and the other bmph located on the opposite side of the central benzene ring. The dihedral angles between the central benzene ring and the three pendant phenoxy rings are 76.71 (14), 67.81 (13) and 70.67 (16)°. In the crystal structure, one bmph is disordered over two sites in a 0.611 (5):0.389 (5) ratio. Some of the methyl H atoms are equally disordered over two sets of sites. Inter­molecular C—H⋯N hydrogen bonding is present in the crystal structure.

## Related literature

For general background, see: Androsov & Neckers (2007 ▶); Blackman (2005 ▶); Fan *et al.* (2003 ▶); Fujita *et al.* (1995 ▶); Li *et al.* (2007 ▶); Zeng & Zimmerman (1997 ▶); Zhao *et al.* (2005 ▶). For related structures, see: Selvanayagam *et al.* (2004 ▶); Cai *et al.* (2004 ▶). For the synthesis, see: Gong *et al.* (2007 ▶); van der Made & van der Made (1993 ▶).
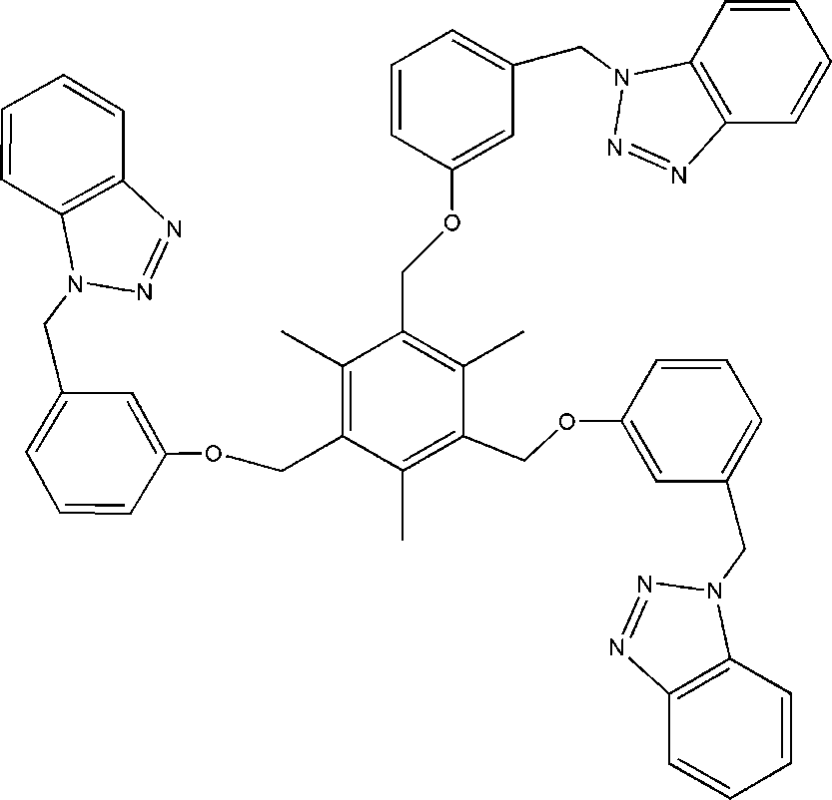


## Experimental

### 

#### Crystal data

C_51_H_45_N_9_O_3_
*M* *_r_* = 831.96
Triclinic, 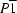

*a* = 11.945 (3) Å
*b* = 13.524 (3) Å
*c* = 13.550 (3) Å
α = 83.913 (4)°
β = 80.629 (4)°
γ = 84.766 (4)°
*V* = 2141.4 (9) Å^3^
*Z* = 2
Mo *K*α radiation
μ = 0.08 mm^−1^
*T* = 295 (2) K
0.23 × 0.19 × 0.18 mm

#### Data collection

Bruker SMART APEXII CCD area-detector diffractometer
Absorption correction: none
16527 measured reflections
7926 independent reflections
3519 reflections with *I* > 2σ(*I*)
*R*_int_ = 0.035

#### Refinement

*R*[*F* ^2^ > 2σ(*F* ^2^)] = 0.057
*wR*(*F* ^2^) = 0.186
*S* = 1.01
7926 reflections
552 parameters
60 restraints
H-atom parameters constrained
Δρ_max_ = 0.15 e Å^−3^
Δρ_min_ = −0.28 e Å^−3^

### 

Data collection: *APEX2* (Bruker, 2004 ▶); cell refinement: *SAINT* (Bruker, 2004 ▶); data reduction: *SAINT*; program(s) used to solve structure: *SHELXS97* (Sheldrick, 2008 ▶); program(s) used to refine structure: *SHELXL97* (Sheldrick, 2008 ▶); molecular graphics: *ORTEP-3 for Windows* (Farrugia, 1997 ▶); software used to prepare material for publication: *WinGX* (Farrugia, 1999 ▶).

## Supplementary Material

Crystal structure: contains datablocks global, I. DOI: 10.1107/S1600536808028845/xu2438sup1.cif

Structure factors: contains datablocks I. DOI: 10.1107/S1600536808028845/xu2438Isup2.hkl

Additional supplementary materials:  crystallographic information; 3D view; checkCIF report

## Figures and Tables

**Table 1:** Hydrogen-bond geometry (Å, °)

*D*—H⋯*A*	*D*—H	H⋯*A*	*D*⋯*A*	*D*—H⋯*A*
C2—H2⋯N8^i^	0.93	2.48	3.376 (7)	163
C27—H27⋯N1^ii^	0.93	2.54	3.453 (7)	166

Symmetry codes: (i) 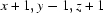; (ii) 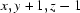.

**Fig. 1.**
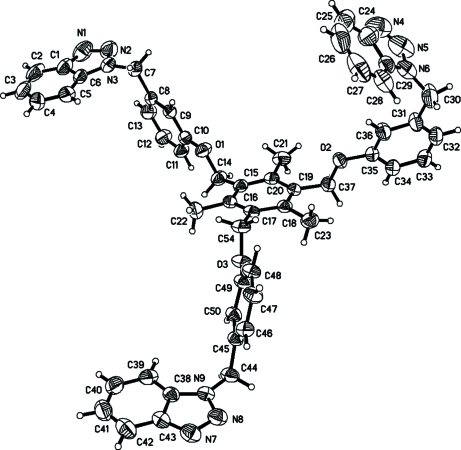
The molecular structure of the title compound with 30% displacement probability ellipsoids.

## supplementary crystallographic information

### Comment

Tripodal ligands based on nitrogen heterocycles have been widely employed in many areas of inorganic chemistry (Blackman, 2005). For example, tripodal ligands with an arene core have been found to be one of the most useful organic building blocks in construction of metal-organic frameworks(MOFs) (Zeng & Zimmerman, 1997; Li *et al.*, 2007). Especially in the case of flexible tripodal ligands such as 1,3,5-tris(4-pyridylmethyl)benzene (Fujita *et al.*, 1995) and 1,3,5-tris(imidazol-1-ylmethyl)-2,4,6-trimethylbenzene (Fan *et al.*, 2003), which have many more possible coordination modes due to their flexibility, and they can adopt different conformations according to geometric requirements of different metal ions (Zhao *et al.*, 2005). However, only a few examples of flexible tripodal benzotriazole ligands are reported (Androsov & Neckers, 2007). Herein we report the crystal structure of the title compound.

A view of the molecular structure of the title compound is given in Fig.1. IN the crystal structure the three 1-(1*H*-benzotriazol-1-ylmethyl)-3-phenyloxy (bmph) groups are bonded to the central benzene ring with an asymmetric arrangement, two bmph located on one side of the central benzene ring and the other bmph located on the opposite side of the central benzene ring. The dihedral angles between each benzotriazole moiety and the phenyloxy benzene rings are 86.1 (2), 91.8 (3) and 109.2 (2)°, respectively, and dihedral angles between mesitylene and phenyloxy benzene rings are 121.0 (2), 70.7 (3) and 112.2 (3)°. The N1-benzotriazole and N7-benzotriazole are approximately parallel to each other with a dihedral angle of 9.4 (2)°. All the bond distances and angles in the structure are within normal ranges, similar to those found in the related compound (Selvanayagam *et al.*, 2004; Cai *et al.*, 2004). Intermolecular C—H···N hydrogen bonding presents in the crystal structure (Table 1).

### Experimental

1,3,5-Tris(bromomethyl)-2,4,6-trimethylbenzene and 1-(benzotriazol-1-ylmethyl)- 3-hydroxybenzene were synthesized according to the reported procedure was (van der Made *et al.*, 1993; Gong *et al.*, 2007). 1,3,5-Tris(bromomethyl)-2,4,6- trimethylbenzene (3 mmol), 1-(benzotriazol-1-ylmethyl)-3-hydroxybenzene (9 mmol) and NaH (27 mmol) were dissolved in dry dioxane (25 ml), then the resultant solution was refluxed for 6 h, removal of solvent resulted in a white powder that was recrystallized from dichloromethane-petroleum ether solution at room temperature to give the desired product as colorless crystals suitable for single-crystal X-ray diffraction (yield 55%; m.p > 573 K).

### Refinement

The N4-containing benzotriazole is disordered over two sites, occupancies were refined and converged to 0.611 (5):0.389 (5). The rigid-group mode was used in refinement for the disordered components, and atomic displacement parameters were constrained for disordered components. H atoms were placed in geometrically idealized positions and treated as riding with C—H = 0.93 (aromatic), 0.96 Å (methyl) and 0.97 Å (methylene), and constrained to ride on their parent atoms with U_iso_(H) = 1.5U_eq_(C) (for methyl) or 1.2U_eq_(C) for others.

### Crystal data

**Table:**

C_51_H_45_N_9_O_3_	*Z* = 2
*M**_r_* = 831.96	*F*(000) = 876
Triclinic, *P*1	*D*_x_ = 1.290 Mg m^−^^3^
Hall symbol: -P 1	Mo *K*α radiation, λ = 0.71073 Å
*a* = 11.945 (3) Å	Cell parameters from 1969 reflections
*b* = 13.524 (3) Å	θ = 2.5–20.4°
*c* = 13.550 (3) Å	µ = 0.08 mm^−^^1^
α = 83.913 (4)°	*T* = 295 K
β = 80.629 (4)°	Block, colourless
γ = 84.766 (4)°	0.23 × 0.19 × 0.18 mm
*V* = 2141.4 (9) Å^3^	

### Data collection

**Table:**

Bruker SMART CCD area-detector diffractometer	3519 reflections with *I* > 2σ(*I*)
Radiation source: fine-focus sealed tube	*R*_int_ = 0.035
graphite	θ_max_ = 25.5°, θ_min_ = 2.5°
φ and ω scans	*h* = −14→14
16527 measured reflections	*k* = −16→16
7926 independent reflections	*l* = −16→16

### Refinement

**Table:**

Refinement on *F*^2^	Primary atom site location: structure-invariant direct methods
Least-squares matrix: full	Secondary atom site location: difference Fourier map
*R*[*F*^2^ > 2σ(*F*^2^)] = 0.057	Hydrogen site location: inferred from neighbouring sites
*wR*(*F*^2^) = 0.186	H-atom parameters constrained
*S* = 1.01	*w* = 1/[σ^2^(*F*_o_^2^) + (0.0806*P*)^2^ + 0.0204*P*] where *P* = (*F*_o_^2^ + 2*F*_c_^2^)/3
7926 reflections	(Δ/σ)_max_ < 0.001
552 parameters	Δρ_max_ = 0.15 e Å^−^^3^
60 restraints	Δρ_min_ = −0.28 e Å^−^^3^

### Special details

**Table:**

Experimental. Analysis found: C 73.85, H 5.29, N 15.37%; requires: C 73.63, H 5.45, N 15.15%. IR data (v_max/ cm^-1^): 3425, 2924, 1593, 1489, 1451, 1257, 1158, 1089, 1009, 781, 747. NMR δ(H) 1.85(6*H*,brs), 2.32(9*H*,s), 4.99(6*H*,s), 5.84(6*H*,s), 6.90(6*H*,d), 6.95 (3*H*,d), 7.26(3*H*,s), 7.35–7.43(9*H*,s), 8.08(3*H*,s). MS-ESI^+^ [m/*z*]: 854.6(*M*+Na).
Geometry. All e.s.d.'s (except the e.s.d. in the dihedral angle between two l.s. planes)are estimated using the full covariance matrix. The cell e.s.d.'s are takeninto account individually in the estimation of e.s.d.'s in distances, anglesand torsion angles; correlations between e.s.d.'s in cell parameters are onlyused when they are defined by crystal symmetry. An approximate (isotropic)treatment of cell e.s.d.'s is used for estimating e.s.d.'s involving l.s. planes.
Refinement. Refinement of *F*^2^ against ALL reflections. The weighted *R*-factor *wR* and goodness of fit *S* are based on *F*^2^, conventional *R*-factors *R* are based on *F*, with *F* set to zero for negative *F*^2^. The threshold expression of *F*^2^ > σ(*F*^2^) is used only for calculating *R*-factors(gt) *etc*. and is not relevant to the choice of reflections for refinement. *R*-factors based on *F*^2^ are statistically about twice as large as those based on *F*, and *R*- factors based on ALL data will be even larger.

### Fractional atomic coordinates and isotropic or equivalent isotropic displacement parameters (Å^2^)

**Table:**

	*x*	*y*	*z*	*U*_iso_*/*U*_eq_	Occ. (<1)
N6	0.8300 (6)	1.4682 (5)	0.5035 (4)	0.0889 (13)	0.611 (5)
N4	0.9689 (5)	1.3866 (4)	0.5597 (4)	0.125 (2)	0.611 (5)
N5	0.8893 (7)	1.4545 (5)	0.5804 (4)	0.121 (2)	0.611 (5)
C24	0.9686 (4)	1.3515 (3)	0.4672 (3)	0.0935 (17)	0.611 (5)
C25	1.0378 (4)	1.2814 (4)	0.4112 (4)	0.114 (2)	0.611 (5)
H25	1.1005	1.2456	0.4338	0.137*	0.611 (5)
C26	1.0059 (5)	1.2696 (4)	0.3202 (4)	0.125 (2)	0.611 (5)
H26	1.0463	1.2209	0.2823	0.150*	0.611 (5)
C27	0.9172 (5)	1.3259 (5)	0.2818 (4)	0.112 (2)	0.611 (5)
H27	0.9030	1.3169	0.2180	0.134*	0.611 (5)
C28	0.8493 (5)	1.3958 (5)	0.3379 (4)	0.0961 (15)	0.611 (5)
H28	0.7888	1.4332	0.3133	0.115*	0.611 (5)
C29	0.8757 (4)	1.4072 (4)	0.4326 (3)	0.0780 (10)	0.611 (5)
C30	0.7351 (8)	1.5351 (7)	0.5177 (6)	0.1372 (18)	0.611 (5)
H30A	0.7104	1.5556	0.4534	0.165*	0.611 (5)
H30B	0.7558	1.5940	0.5436	0.165*	0.611 (5)
C1	0.8340 (3)	0.2950 (3)	0.9635 (4)	0.0977 (12)	
N6'	0.8453 (10)	1.4436 (8)	0.5142 (7)	0.0889 (13)	0.389 (5)
N4'	0.9868 (9)	1.3395 (7)	0.5248 (6)	0.125 (2)	0.389 (5)
N5'	0.9219 (11)	1.4049 (8)	0.5727 (6)	0.121 (2)	0.389 (5)
C24'	0.9586 (6)	1.3312 (5)	0.4307 (6)	0.0935 (17)	0.389 (5)
C25'	1.0041 (7)	1.2740 (6)	0.3510 (6)	0.114 (2)	0.389 (5)
H25'	1.0673	1.2288	0.3533	0.137*	0.389 (5)
C26'	0.9478 (8)	1.2901 (6)	0.2689 (6)	0.125 (2)	0.389 (5)
H26'	0.9718	1.2510	0.2159	0.150*	0.389 (5)
C27'	0.8575 (9)	1.3611 (7)	0.2605 (6)	0.112 (2)	0.389 (5)
H27'	0.8262	1.3709	0.2014	0.134*	0.389 (5)
C28'	0.8132 (8)	1.4179 (7)	0.3401 (7)	0.0961 (15)	0.389 (5)
H28'	0.7520	1.4649	0.3357	0.115*	0.389 (5)
C29'	0.8647 (7)	1.4011 (6)	0.4266 (6)	0.0780 (10)	0.389 (5)
C30'	0.7393 (14)	1.5328 (12)	0.5253 (10)	0.1372 (18)	0.389 (5)
H30C	0.7623	1.5882	0.5553	0.165*	0.389 (5)
H30D	0.7209	1.5571	0.4594	0.165*	0.389 (5)
O1	0.52998 (17)	0.80350 (16)	0.79301 (16)	0.0819 (7)	
O2	0.50213 (17)	1.26023 (15)	0.64934 (17)	0.0810 (6)	
O3	0.22369 (16)	0.99186 (17)	0.46236 (15)	0.0804 (7)	
N1	0.8768 (4)	0.3362 (4)	1.0342 (3)	0.1399 (15)	
N2	0.8791 (3)	0.4321 (4)	1.0098 (3)	0.1267 (13)	
N3	0.8385 (2)	0.4531 (2)	0.9210 (3)	0.0888 (9)	
N7	−0.1788 (3)	0.9250 (3)	0.0660 (2)	0.0910 (9)	
N8	−0.1573 (2)	0.9894 (2)	0.1239 (2)	0.0851 (8)	
N9	−0.1164 (2)	0.9398 (2)	0.20454 (19)	0.0675 (7)	
C2	0.8185 (5)	0.1936 (4)	0.9601 (5)	0.140 (2)	
H2	0.8392	0.1431	1.0073	0.168*	
C3	0.7706 (5)	0.1785 (5)	0.8811 (7)	0.172 (4)	
H3	0.7557	0.1133	0.8751	0.207*	
C4	0.7413 (4)	0.2490 (5)	0.8086 (5)	0.145 (2)	
H4	0.7080	0.2303	0.7566	0.174*	
C5	0.7601 (3)	0.3466 (3)	0.8110 (3)	0.1000 (12)	
H5	0.7419	0.3956	0.7614	0.120*	
C6	0.8075 (3)	0.3680 (3)	0.8913 (3)	0.0755 (9)	
C7	0.8255 (3)	0.5545 (2)	0.8760 (3)	0.1036 (13)	
H7A	0.8435	0.5545	0.8035	0.124*	
H7B	0.8799	0.5934	0.8976	0.124*	
C8	0.7072 (3)	0.6044 (2)	0.9025 (3)	0.0732 (9)	
C9	0.6679 (3)	0.6814 (2)	0.8388 (2)	0.0693 (8)	
H9	0.7135	0.7014	0.7789	0.083*	
C10	0.5610 (3)	0.7291 (2)	0.8634 (2)	0.0659 (8)	
C11	0.4925 (3)	0.7001 (2)	0.9517 (2)	0.0761 (9)	
H11	0.4200	0.7313	0.9677	0.091*	
C12	0.5323 (3)	0.6247 (3)	1.0157 (3)	0.0876 (11)	
H12	0.4870	0.6057	1.0761	0.105*	
C13	0.6384 (3)	0.5767 (3)	0.9918 (3)	0.0880 (11)	
H13	0.6640	0.5254	1.0359	0.106*	
C14	0.4173 (3)	0.8535 (2)	0.8129 (2)	0.0791 (10)	
H14A	0.4053	0.8782	0.8787	0.095*	
H14B	0.3602	0.8072	0.8117	0.095*	
C15	0.4073 (2)	0.9381 (2)	0.7340 (2)	0.0626 (8)	
C16	0.3786 (2)	0.9206 (2)	0.6416 (3)	0.0638 (8)	
C17	0.3626 (2)	1.0009 (2)	0.5703 (2)	0.0610 (8)	
C18	0.3663 (2)	1.0990 (2)	0.5952 (2)	0.0627 (8)	
C19	0.3975 (2)	1.1159 (2)	0.6862 (2)	0.0602 (8)	
C20	0.4227 (2)	1.0347 (2)	0.7532 (2)	0.0618 (8)	
C21	0.4674 (3)	1.0522 (3)	0.8487 (3)	0.0912 (11)	
H21A	0.4045	1.0677	0.8999	0.137*	
H21B	0.5154	1.1067	0.8349	0.137*	
H21C	0.5103	0.9930	0.8714	0.137*	
C22	0.3609 (3)	0.8167 (2)	0.6193 (3)	0.0963 (11)	
H22A	0.3750	0.7702	0.6753	0.145*	0.50
H22B	0.4125	0.7998	0.5604	0.145*	0.50
H22C	0.2840	0.8141	0.6079	0.145*	0.50
H22D	0.3394	0.8192	0.5538	0.145*	0.50
H22E	0.3019	0.7896	0.6687	0.145*	0.50
H22F	0.4303	0.7752	0.6211	0.145*	0.50
C23	0.3347 (3)	1.1869 (3)	0.5227 (3)	0.0932 (11)	
H23A	0.3152	1.1628	0.4640	0.140*	0.50
H23B	0.3981	1.2274	0.5035	0.140*	0.50
H23C	0.2706	1.2262	0.5548	0.140*	0.50
H23D	0.3407	1.2481	0.5509	0.140*	0.50
H23E	0.2579	1.1835	0.5113	0.140*	0.50
H23F	0.3853	1.1847	0.4601	0.140*	0.50
C31	0.6374 (3)	1.4917 (3)	0.5896 (3)	0.0865 (10)	
C32	0.5693 (4)	1.5536 (3)	0.6508 (3)	0.0880 (11)	
H32	0.5862	1.6194	0.6505	0.106*	
C33	0.4763 (3)	1.5194 (3)	0.7124 (3)	0.0913 (11)	
H33	0.4304	1.5621	0.7541	0.110*	
C34	0.4491 (3)	1.4217 (3)	0.7139 (3)	0.0795 (9)	
H34	0.3852	1.3991	0.7560	0.095*	
C35	0.5171 (3)	1.3589 (2)	0.6528 (2)	0.0692 (8)	
C36	0.6102 (3)	1.3938 (3)	0.5901 (3)	0.0850 (10)	
H36	0.6555	1.3516	0.5475	0.102*	
C37	0.4026 (3)	1.2207 (2)	0.7102 (3)	0.0768 (9)	
H37A	0.3347	1.2608	0.6958	0.092*	
H37B	0.4074	1.2217	0.7809	0.092*	
C38	−0.1114 (2)	0.8400 (3)	0.1982 (2)	0.0667 (8)	
C39	−0.0778 (3)	0.7585 (3)	0.2608 (3)	0.0853 (10)	
H39	−0.0506	0.7654	0.3200	0.102*	
C40	−0.0876 (3)	0.6666 (3)	0.2290 (4)	0.1050 (13)	
H40	−0.0656	0.6092	0.2675	0.126*	
C41	−0.1294 (4)	0.6577 (4)	0.1413 (4)	0.1147 (15)	
H41	−0.1352	0.5942	0.1232	0.138*	
C42	−0.1626 (4)	0.7386 (4)	0.0797 (3)	0.1080 (13)	
H42	−0.1908	0.7312	0.0211	0.130*	
C43	−0.1521 (3)	0.8316 (3)	0.1091 (3)	0.0754 (9)	
C44	−0.0932 (2)	0.9947 (3)	0.2840 (2)	0.0778 (9)	
H44A	−0.1220	0.9599	0.3482	0.093*	
H44B	−0.1345	1.0598	0.2797	0.093*	
C45	0.0306 (2)	1.0086 (2)	0.2814 (2)	0.0619 (8)	
C46	0.0954 (3)	1.0492 (2)	0.1951 (2)	0.0717 (9)	
H46	0.0644	1.0635	0.1361	0.086*	
C47	0.2056 (3)	1.0681 (2)	0.1973 (2)	0.0779 (9)	
H47	0.2492	1.0947	0.1390	0.094*	
C48	0.2531 (3)	1.0488 (2)	0.2836 (2)	0.0716 (9)	
H48	0.3280	1.0626	0.2839	0.086*	
C49	0.1888 (2)	1.0089 (2)	0.3698 (2)	0.0618 (8)	
C50	0.0784 (2)	0.9872 (2)	0.3678 (2)	0.0641 (8)	
H50	0.0360	0.9578	0.4253	0.077*	
C54	0.3440 (2)	0.9854 (3)	0.4660 (2)	0.0791 (10)	
H54A	0.3801	1.0357	0.4181	0.095*	
H54B	0.3779	0.9204	0.4484	0.095*	

### Atomic displacement parameters (Å^2^)

**Table:**

	*U*^11^	*U*^22^	*U*^33^	*U*^12^	*U*^13^	*U*^23^
N6	0.098 (3)	0.083 (4)	0.089 (3)	−0.033 (2)	−0.024 (2)	0.011 (2)
N4	0.123 (4)	0.130 (5)	0.124 (5)	−0.022 (4)	−0.034 (4)	0.015 (4)
N5	0.128 (5)	0.137 (6)	0.102 (3)	−0.037 (4)	−0.031 (3)	0.009 (3)
C24	0.089 (3)	0.086 (3)	0.102 (4)	−0.014 (3)	−0.011 (3)	0.007 (3)
C25	0.103 (4)	0.089 (3)	0.140 (6)	0.004 (3)	0.002 (4)	−0.002 (4)
C26	0.121 (5)	0.087 (4)	0.159 (6)	−0.015 (4)	0.003 (4)	−0.012 (4)
C27	0.121 (6)	0.100 (5)	0.115 (4)	−0.034 (4)	−0.003 (4)	−0.015 (3)
C28	0.094 (5)	0.086 (4)	0.111 (3)	−0.019 (3)	−0.018 (3)	−0.006 (3)
C29	0.084 (2)	0.070 (2)	0.084 (2)	−0.0271 (19)	−0.020 (2)	0.0054 (19)
C30	0.142 (4)	0.069 (3)	0.184 (5)	−0.019 (3)	0.037 (4)	−0.024 (3)
C1	0.088 (3)	0.082 (3)	0.111 (3)	0.019 (2)	−0.007 (2)	0.011 (3)
N6'	0.098 (3)	0.083 (4)	0.089 (3)	−0.033 (2)	−0.024 (2)	0.011 (2)
N4'	0.123 (4)	0.130 (5)	0.124 (5)	−0.022 (4)	−0.034 (4)	0.015 (4)
N5'	0.128 (5)	0.137 (6)	0.102 (3)	−0.037 (4)	−0.031 (3)	0.009 (3)
C24'	0.089 (3)	0.086 (3)	0.102 (4)	−0.014 (3)	−0.011 (3)	0.007 (3)
C25'	0.103 (4)	0.089 (3)	0.140 (6)	0.004 (3)	0.002 (4)	−0.002 (4)
C26'	0.121 (5)	0.087 (4)	0.159 (6)	−0.015 (4)	0.003 (4)	−0.012 (4)
C27'	0.121 (6)	0.100 (5)	0.115 (4)	−0.034 (4)	−0.003 (4)	−0.015 (3)
C28'	0.094 (5)	0.086 (4)	0.111 (3)	−0.019 (3)	−0.018 (3)	−0.006 (3)
C29'	0.084 (2)	0.070 (2)	0.084 (2)	−0.0271 (19)	−0.020 (2)	0.0054 (19)
C30'	0.142 (4)	0.069 (3)	0.184 (5)	−0.019 (3)	0.037 (4)	−0.024 (3)
O1	0.0603 (14)	0.0778 (15)	0.0961 (16)	0.0050 (11)	0.0009 (12)	0.0168 (13)
O2	0.0683 (14)	0.0627 (15)	0.1113 (17)	−0.0157 (11)	0.0020 (13)	−0.0201 (12)
O3	0.0506 (13)	0.1262 (19)	0.0672 (14)	−0.0247 (12)	−0.0066 (11)	−0.0100 (12)
N1	0.151 (4)	0.135 (4)	0.128 (3)	0.040 (3)	−0.043 (3)	0.005 (3)
N2	0.128 (3)	0.123 (3)	0.140 (3)	0.017 (3)	−0.058 (3)	−0.030 (3)
N3	0.083 (2)	0.067 (2)	0.116 (3)	0.0078 (15)	−0.0247 (19)	−0.0026 (18)
N7	0.096 (2)	0.106 (3)	0.075 (2)	−0.0250 (19)	−0.0233 (17)	0.0031 (19)
N8	0.078 (2)	0.091 (2)	0.087 (2)	−0.0187 (16)	−0.0211 (17)	0.0105 (18)
N9	0.0588 (16)	0.079 (2)	0.0680 (17)	−0.0132 (13)	−0.0170 (13)	−0.0058 (15)
C2	0.091 (4)	0.085 (4)	0.208 (6)	0.023 (3)	0.026 (4)	0.052 (4)
C3	0.087 (4)	0.075 (4)	0.334 (12)	0.017 (3)	0.019 (5)	−0.021 (6)
C4	0.096 (4)	0.127 (5)	0.224 (7)	−0.001 (3)	−0.015 (4)	−0.098 (5)
C5	0.083 (3)	0.104 (3)	0.116 (3)	0.000 (2)	−0.016 (2)	−0.028 (3)
C6	0.066 (2)	0.058 (2)	0.096 (3)	0.0031 (17)	−0.004 (2)	0.002 (2)
C7	0.077 (3)	0.060 (2)	0.166 (4)	−0.0003 (18)	−0.009 (2)	0.003 (2)
C8	0.063 (2)	0.0493 (19)	0.106 (3)	−0.0070 (16)	−0.011 (2)	−0.0034 (18)
C9	0.060 (2)	0.0561 (19)	0.089 (2)	−0.0108 (16)	−0.0023 (17)	−0.0028 (17)
C10	0.061 (2)	0.0575 (19)	0.077 (2)	−0.0105 (16)	−0.0081 (18)	0.0016 (17)
C11	0.061 (2)	0.073 (2)	0.086 (2)	0.0013 (17)	0.0001 (19)	0.0042 (19)
C12	0.074 (2)	0.088 (3)	0.091 (3)	−0.003 (2)	0.000 (2)	0.018 (2)
C13	0.081 (3)	0.079 (2)	0.099 (3)	−0.007 (2)	−0.017 (2)	0.018 (2)
C14	0.059 (2)	0.079 (2)	0.089 (2)	0.0016 (17)	0.0027 (17)	0.0127 (19)
C15	0.0456 (17)	0.065 (2)	0.073 (2)	−0.0032 (14)	−0.0016 (15)	0.0032 (17)
C16	0.0449 (17)	0.063 (2)	0.083 (2)	−0.0096 (14)	−0.0028 (16)	−0.0085 (18)
C17	0.0380 (16)	0.080 (2)	0.066 (2)	−0.0111 (14)	−0.0068 (14)	−0.0110 (18)
C18	0.0421 (17)	0.068 (2)	0.075 (2)	−0.0090 (14)	−0.0055 (15)	0.0083 (17)
C19	0.0480 (17)	0.060 (2)	0.073 (2)	−0.0074 (14)	−0.0072 (15)	−0.0085 (17)
C20	0.0448 (17)	0.077 (2)	0.063 (2)	−0.0041 (15)	−0.0049 (14)	−0.0082 (17)
C21	0.073 (2)	0.117 (3)	0.088 (3)	−0.002 (2)	−0.021 (2)	−0.019 (2)
C22	0.087 (3)	0.073 (2)	0.131 (3)	−0.0124 (19)	−0.011 (2)	−0.026 (2)
C23	0.075 (2)	0.093 (3)	0.108 (3)	−0.0092 (19)	−0.024 (2)	0.023 (2)
C31	0.102 (3)	0.062 (2)	0.095 (3)	−0.021 (2)	−0.001 (2)	−0.009 (2)
C32	0.106 (3)	0.063 (2)	0.098 (3)	−0.006 (2)	−0.021 (2)	−0.012 (2)
C33	0.101 (3)	0.072 (3)	0.102 (3)	0.003 (2)	−0.015 (2)	−0.020 (2)
C34	0.079 (2)	0.068 (2)	0.092 (2)	−0.0046 (19)	−0.012 (2)	−0.0094 (19)
C35	0.070 (2)	0.058 (2)	0.084 (2)	−0.0053 (17)	−0.0175 (18)	−0.0135 (18)
C36	0.083 (3)	0.074 (2)	0.096 (3)	−0.0157 (19)	0.006 (2)	−0.0192 (19)
C37	0.061 (2)	0.068 (2)	0.099 (2)	−0.0078 (16)	−0.0021 (18)	−0.0105 (18)
C38	0.0533 (19)	0.073 (2)	0.074 (2)	−0.0151 (16)	−0.0047 (16)	−0.0031 (18)
C39	0.064 (2)	0.098 (3)	0.092 (3)	−0.005 (2)	−0.0127 (19)	0.004 (2)
C40	0.081 (3)	0.084 (3)	0.146 (4)	−0.002 (2)	−0.012 (3)	−0.005 (3)
C41	0.096 (3)	0.094 (4)	0.156 (5)	−0.021 (3)	0.000 (3)	−0.042 (3)
C42	0.105 (3)	0.120 (4)	0.109 (3)	−0.030 (3)	−0.012 (3)	−0.042 (3)
C43	0.071 (2)	0.090 (3)	0.068 (2)	−0.0172 (19)	−0.0101 (18)	−0.012 (2)
C44	0.053 (2)	0.101 (3)	0.085 (2)	−0.0101 (17)	−0.0092 (17)	−0.032 (2)
C45	0.0502 (18)	0.069 (2)	0.069 (2)	−0.0087 (15)	−0.0041 (16)	−0.0224 (16)
C46	0.061 (2)	0.085 (2)	0.070 (2)	−0.0075 (17)	−0.0101 (17)	−0.0101 (17)
C47	0.064 (2)	0.100 (3)	0.069 (2)	−0.0247 (18)	−0.0005 (18)	−0.0050 (18)
C48	0.0545 (19)	0.094 (2)	0.068 (2)	−0.0223 (17)	−0.0070 (17)	−0.0080 (18)
C49	0.0497 (18)	0.077 (2)	0.060 (2)	−0.0137 (15)	−0.0023 (15)	−0.0155 (16)
C50	0.0510 (18)	0.078 (2)	0.064 (2)	−0.0156 (15)	0.0026 (15)	−0.0175 (16)
C54	0.049 (2)	0.114 (3)	0.076 (2)	−0.0111 (17)	−0.0075 (16)	−0.0170 (19)

### Geometric parameters (Å, °)

**Table:**

N6—N5	1.3401	C11—H11	0.9300
N6—C29	1.3481	C12—C13	1.376 (4)
N6—C30	1.3851	C12—H12	0.9300
N4—N5	1.2782	C13—H13	0.9300
N4—C24	1.3871	C14—C15	1.492 (4)
C24—C29	1.4007	C14—H14A	0.9700
C24—C25	1.4014	C14—H14B	0.9700
C25—C26	1.3775	C15—C20	1.392 (4)
C25—H25	0.9300	C15—C16	1.399 (4)
C26—C27	1.3855	C16—C17	1.396 (4)
C26—H26	0.9300	C16—C22	1.508 (4)
C27—C28	1.3936	C17—C18	1.410 (4)
C27—H27	0.9300	C17—C54	1.505 (4)
C28—C29	1.3972	C18—C19	1.391 (4)
C28—H28	0.9300	C18—C23	1.521 (4)
C30—C31	1.513 (13)	C19—C20	1.392 (4)
C30—H30A	0.9700	C19—C37	1.497 (4)
C30—H30B	0.9700	C20—C21	1.526 (4)
C1—N1	1.347 (5)	C21—H21A	0.9600
C1—C6	1.367 (5)	C21—H21B	0.9600
C1—C2	1.407 (7)	C21—H21C	0.9600
N6'—N5'	1.3420	C22—H22A	0.9600
N6'—C29'	1.3500	C22—H22B	0.9600
N6'—C30'	1.6672	C22—H22C	0.9600
N4'—N5'	1.2800	C22—H22D	0.9600
N4'—C24'	1.3891	C22—H22E	0.9600
C24'—C29'	1.4027	C22—H22F	0.9600
C24'—C25'	1.4034	C23—H23A	0.9600
C25'—C26'	1.3795	C23—H23B	0.9600
C25'—H25'	0.9300	C23—H23C	0.9600
C26'—C27'	1.3875	C23—H23D	0.9600
C26'—H26'	0.9300	C23—H23E	0.9600
C27'—C28'	1.3956	C23—H23F	0.9600
C27'—H27'	0.9300	C31—C32	1.362 (5)
C28'—C29'	1.3992	C31—C36	1.390 (4)
C28'—H28'	0.9300	C32—C33	1.363 (5)
C30'—C31	1.49 (2)	C32—H32	0.9300
C30'—H30C	0.9700	C33—C34	1.386 (4)
C30'—H30D	0.9700	C33—H33	0.9300
O1—C10	1.380 (3)	C34—C35	1.368 (4)
O1—C14	1.447 (3)	C34—H34	0.9300
O2—C35	1.369 (3)	C35—C36	1.373 (4)
O2—C37	1.443 (3)	C36—H36	0.9300
O3—C49	1.375 (3)	C37—H37A	0.9700
O3—C54	1.441 (3)	C37—H37B	0.9700
N1—N2	1.306 (5)	C38—C39	1.389 (4)
N2—N3	1.363 (4)	C38—C43	1.392 (4)
N3—C6	1.360 (4)	C39—C40	1.379 (5)
N3—C7	1.446 (4)	C39—H39	0.9300
N7—N8	1.301 (4)	C40—C41	1.382 (6)
N7—C43	1.367 (4)	C40—H40	0.9300
N8—N9	1.358 (3)	C41—C42	1.374 (6)
N9—C38	1.356 (4)	C41—H41	0.9300
N9—C44	1.447 (4)	C42—C43	1.381 (5)
C2—C3	1.335 (8)	C42—H42	0.9300
C2—H2	0.9300	C44—C45	1.502 (4)
C3—C4	1.360 (8)	C44—H44A	0.9700
C3—H3	0.9300	C44—H44B	0.9700
C4—C5	1.364 (6)	C45—C50	1.377 (4)
C4—H4	0.9300	C45—C46	1.384 (4)
C5—C6	1.373 (5)	C46—C47	1.369 (4)
C5—H5	0.9300	C46—H46	0.9300
C7—C8	1.514 (4)	C47—C48	1.372 (4)
C7—H7A	0.9700	C47—H47	0.9300
C7—H7B	0.9700	C48—C49	1.377 (4)
C8—C9	1.379 (4)	C48—H48	0.9300
C8—C13	1.385 (4)	C49—C50	1.381 (4)
C9—C10	1.383 (4)	C50—H50	0.9300
C9—H9	0.9300	C54—H54A	0.9700
C10—C11	1.377 (4)	C54—H54B	0.9700
C11—C12	1.369 (4)		
			
N5—N6—C29	109.9	C17—C18—C23	119.8 (3)
N5—N6—C30	114.4	C18—C19—C20	119.2 (3)
C29—N6—C30	135.5	C18—C19—C37	119.4 (3)
N5—N4—C24	112.7	C20—C19—C37	121.4 (3)
N4—N5—N6	107.3	C19—C20—C15	120.7 (3)
N4—C24—C29	102.9	C19—C20—C21	119.4 (3)
N4—C24—C25	134.3	C15—C20—C21	119.9 (3)
C29—C24—C25	122.8	C20—C21—H21A	109.5
C26—C25—C24	114.8	C20—C21—H21B	109.5
C26—C25—H25	122.6	H21A—C21—H21B	109.5
C24—C25—H25	122.6	C20—C21—H21C	109.5
C25—C26—C27	124.1	H21A—C21—H21C	109.5
C25—C26—H26	118.0	H21B—C21—H21C	109.5
C27—C26—H26	118.0	C16—C22—H22A	109.5
C26—C27—C28	120.5	C16—C22—H22B	109.5
C26—C27—H27	119.7	H22A—C22—H22B	109.5
C28—C27—H27	119.7	C16—C22—H22C	109.5
C27—C28—C29	117.3	H22A—C22—H22C	109.5
C27—C28—H28	121.4	H22B—C22—H22C	109.5
C29—C28—H28	121.4	C16—C22—H22D	109.5
N6—C29—C28	132.4	H22A—C22—H22D	141.1
N6—C29—C24	107.2	H22B—C22—H22D	56.3
C28—C29—C24	120.4	H22C—C22—H22D	56.3
N6—C30—C31	112.7 (4)	C16—C22—H22E	109.5
N6—C30—H30A	109.0	H22A—C22—H22E	56.3
C31—C30—H30A	109.0	H22B—C22—H22E	141.1
N6—C30—H30B	109.0	H22C—C22—H22E	56.3
C31—C30—H30B	109.0	H22D—C22—H22E	109.5
H30A—C30—H30B	107.8	C16—C22—H22F	109.5
N1—C1—C6	109.3 (4)	H22A—C22—H22F	56.3
N1—C1—C2	127.4 (5)	H22B—C22—H22F	56.3
C6—C1—C2	123.4 (5)	H22C—C22—H22F	141.1
N5'—N6'—C29'	109.9	H22D—C22—H22F	109.5
N5'—N6'—C30'	134.1	H22E—C22—H22F	109.5
C29'—N6'—C30'	115.9	C18—C23—H23A	109.5
N5'—N4'—C24'	112.7	C18—C23—H23B	109.5
N4'—N5'—N6'	107.3	H23A—C23—H23B	109.5
N4'—C24'—C29'	102.9	C18—C23—H23C	109.5
N4'—C24'—C25'	134.3	H23A—C23—H23C	109.5
C29'—C24'—C25'	122.8	H23B—C23—H23C	109.5
C26'—C25'—C24'	114.8	C18—C23—H23D	109.5
C26'—C25'—H25'	122.6	H23A—C23—H23D	141.1
C24'—C25'—H25'	122.6	H23B—C23—H23D	56.3
C25'—C26'—C27'	124.1	H23C—C23—H23D	56.3
C25'—C26'—H26'	118.0	C18—C23—H23E	109.5
C27'—C26'—H26'	118.0	H23A—C23—H23E	56.3
C26'—C27'—C28'	120.5	H23B—C23—H23E	141.1
C26'—C27'—H27'	119.7	H23C—C23—H23E	56.3
C28'—C27'—H27'	119.7	H23D—C23—H23E	109.5
C27'—C28'—C29'	117.3	C18—C23—H23F	109.5
C27'—C28'—H28'	121.3	H23A—C23—H23F	56.3
C29'—C28'—H28'	121.3	H23B—C23—H23F	56.3
N6'—C29'—C28'	132.4	H23C—C23—H23F	141.1
N6'—C29'—C24'	107.2	H23D—C23—H23F	109.5
C28'—C29'—C24'	120.4	H23E—C23—H23F	109.5
C31—C30'—N6'	109.4 (7)	C32—C31—C36	119.3 (3)
C31—C30'—H30C	109.8	C32—C31—C30'	117.7 (5)
N6'—C30'—H30C	109.8	C36—C31—C30'	123.0 (5)
C31—C30'—H30D	109.8	C32—C31—C30	118.0 (4)
N6'—C30'—H30D	109.8	C36—C31—C30	122.5 (4)
H30C—C30'—H30D	108.2	C31—C32—C33	120.1 (4)
C10—O1—C14	117.7 (2)	C31—C32—H32	120.0
C35—O2—C37	117.5 (2)	C33—C32—H32	120.0
C49—O3—C54	118.1 (2)	C32—C33—C34	121.0 (4)
N2—N1—C1	108.9 (4)	C32—C33—H33	119.5
N1—N2—N3	107.8 (4)	C34—C33—H33	119.5
C6—N3—N2	109.4 (3)	C35—C34—C33	119.4 (3)
C6—N3—C7	129.3 (4)	C35—C34—H34	120.3
N2—N3—C7	121.2 (4)	C33—C34—H34	120.3
N8—N7—C43	108.0 (3)	C34—C35—O2	125.3 (3)
N7—N8—N9	109.0 (3)	C34—C35—C36	119.6 (3)
C38—N9—N8	110.1 (3)	O2—C35—C36	115.1 (3)
C38—N9—C44	130.1 (3)	C35—C36—C31	120.7 (3)
N8—N9—C44	119.7 (3)	C35—C36—H36	119.6
C3—C2—C1	111.9 (6)	C31—C36—H36	119.6
C3—C2—H2	124.0	O2—C37—C19	108.0 (2)
C1—C2—H2	124.0	O2—C37—H37A	110.1
C2—C3—C4	126.6 (7)	C19—C37—H37A	110.1
C2—C3—H3	116.7	O2—C37—H37B	110.1
C4—C3—H3	116.7	C19—C37—H37B	110.1
C3—C4—C5	120.8 (6)	H37A—C37—H37B	108.4
C3—C4—H4	119.6	N9—C38—C39	132.6 (3)
C5—C4—H4	119.6	N9—C38—C43	104.0 (3)
C4—C5—C6	115.8 (5)	C39—C38—C43	123.4 (4)
C4—C5—H5	122.1	C40—C39—C38	115.3 (4)
C6—C5—H5	122.1	C40—C39—H39	122.3
N3—C6—C1	104.6 (4)	C38—C39—H39	122.3
N3—C6—C5	134.0 (4)	C39—C40—C41	121.5 (4)
C1—C6—C5	121.4 (4)	C39—C40—H40	119.2
N3—C7—C8	113.7 (3)	C41—C40—H40	119.2
N3—C7—H7A	108.8	C42—C41—C40	122.9 (4)
C8—C7—H7A	108.8	C42—C41—H41	118.6
N3—C7—H7B	108.8	C40—C41—H41	118.6
C8—C7—H7B	108.8	C41—C42—C43	116.7 (4)
H7A—C7—H7B	107.7	C41—C42—H42	121.6
C9—C8—C13	118.8 (3)	C43—C42—H42	121.6
C9—C8—C7	119.9 (3)	N7—C43—C42	130.9 (4)
C13—C8—C7	121.2 (3)	N7—C43—C38	109.0 (3)
C8—C9—C10	120.4 (3)	C42—C43—C38	120.1 (4)
C8—C9—H9	119.8	N9—C44—C45	114.2 (2)
C10—C9—H9	119.8	N9—C44—H44A	108.7
C11—C10—O1	124.3 (3)	C45—C44—H44A	108.7
C11—C10—C9	120.5 (3)	N9—C44—H44B	108.7
O1—C10—C9	115.2 (3)	C45—C44—H44B	108.7
C12—C11—C10	119.1 (3)	H44A—C44—H44B	107.6
C12—C11—H11	120.4	C50—C45—C46	119.3 (3)
C10—C11—H11	120.4	C50—C45—C44	119.6 (3)
C11—C12—C13	120.9 (3)	C46—C45—C44	120.9 (3)
C11—C12—H12	119.6	C47—C46—C45	119.5 (3)
C13—C12—H12	119.6	C47—C46—H46	120.3
C12—C13—C8	120.3 (3)	C45—C46—H46	120.3
C12—C13—H13	119.8	C46—C47—C48	121.5 (3)
C8—C13—H13	119.8	C46—C47—H47	119.2
O1—C14—C15	108.5 (2)	C48—C47—H47	119.2
O1—C14—H14A	110.0	C47—C48—C49	119.2 (3)
C15—C14—H14A	110.0	C47—C48—H48	120.4
O1—C14—H14B	110.0	C49—C48—H48	120.4
C15—C14—H14B	110.0	O3—C49—C48	125.0 (3)
H14A—C14—H14B	108.4	O3—C49—C50	115.2 (3)
C20—C15—C16	120.1 (3)	C48—C49—C50	119.7 (3)
C20—C15—C14	120.2 (3)	C45—C50—C49	120.7 (3)
C16—C15—C14	119.7 (3)	C45—C50—H50	119.7
C17—C16—C15	119.4 (3)	C49—C50—H50	119.7
C17—C16—C22	119.5 (3)	O3—C54—C17	109.3 (2)
C15—C16—C22	121.0 (3)	O3—C54—H54A	109.8
C16—C17—C18	119.7 (3)	C17—C54—H54A	109.8
C16—C17—C54	121.5 (3)	O3—C54—H54B	109.8
C18—C17—C54	118.8 (3)	C17—C54—H54B	109.8
C19—C18—C17	120.4 (3)	H54A—C54—H54B	108.3
C19—C18—C23	119.8 (3)		
			
C24—N4—N5—N6	0.8	O1—C14—C15—C16	83.8 (3)
C29—N6—N5—N4	−0.7	C20—C15—C16—C17	−1.6 (4)
C30—N6—N5—N4	175.6	C14—C15—C16—C17	176.2 (2)
N5—N4—C24—C29	−0.5	C20—C15—C16—C22	−179.7 (3)
N5—N4—C24—C25	178.1	C14—C15—C16—C22	−1.9 (4)
N4—C24—C25—C26	−179.8	C15—C16—C17—C18	−5.3 (4)
C29—C24—C25—C26	−1.4	C22—C16—C17—C18	172.8 (3)
C24—C25—C26—C27	3.9	C15—C16—C17—C54	173.4 (2)
C25—C26—C27—C28	−3.9	C22—C16—C17—C54	−8.5 (4)
C26—C27—C28—C29	1.0	C16—C17—C18—C19	6.8 (4)
N5—N6—C29—C28	−176.8	C54—C17—C18—C19	−171.9 (2)
C30—N6—C29—C28	7.9	C16—C17—C18—C23	−172.5 (3)
N5—N6—C29—C24	0.4	C54—C17—C18—C23	8.9 (4)
C30—N6—C29—C24	−174.8	C17—C18—C19—C20	−1.3 (4)
C27—C28—C29—N6	178.3	C23—C18—C19—C20	177.9 (3)
C27—C28—C29—C24	1.4	C17—C18—C19—C37	179.0 (2)
N4—C24—C29—N6	0.1	C23—C18—C19—C37	−1.7 (4)
C25—C24—C29—N6	−178.8	C18—C19—C20—C15	−5.7 (4)
N4—C24—C29—C28	177.7	C37—C19—C20—C15	174.0 (3)
C25—C24—C29—C28	−1.2	C18—C19—C20—C21	174.4 (3)
N5—N6—C30—C31	−75.3 (4)	C37—C19—C20—C21	−5.9 (4)
C29—N6—C30—C31	99.7 (4)	C16—C15—C20—C19	7.2 (4)
C24'—N4'—N5'—N6'	0.8	C14—C15—C20—C19	−170.6 (3)
C29'—N6'—N5'—N4'	−0.7	C16—C15—C20—C21	−172.9 (3)
C30'—N6'—N5'—N4'	−177.7	C14—C15—C20—C21	9.3 (4)
N5'—N4'—C24'—C29'	−0.5	N6'—C30'—C31—C32	145.2 (3)
N5'—N4'—C24'—C25'	178.1	N6'—C30'—C31—C36	−34.2 (7)
N4'—C24'—C25'—C26'	−179.8	N6'—C30'—C31—C30	−120 (8)
C29'—C24'—C25'—C26'	−1.4	N6—C30—C31—C32	145.2 (3)
C24'—C25'—C26'—C27'	4.0	N6—C30—C31—C36	−39.5 (5)
C25'—C26'—C27'—C28'	−3.9	N6—C30—C31—C30'	58 (7)
C26'—C27'—C28'—C29'	1.0	C36—C31—C32—C33	0.7 (6)
N5'—N6'—C29'—C28'	−176.8	C30'—C31—C32—C33	−178.8 (6)
C30'—N6'—C29'—C28'	0.8	C30—C31—C32—C33	176.1 (4)
N5'—N6'—C29'—C24'	0.4	C31—C32—C33—C34	−0.2 (6)
C30'—N6'—C29'—C24'	178.0	C32—C33—C34—C35	0.3 (5)
C27'—C28'—C29'—N6'	178.3	C33—C34—C35—O2	177.7 (3)
C27'—C28'—C29'—C24'	1.4	C33—C34—C35—C36	−0.9 (5)
N4'—C24'—C29'—N6'	0.1	C37—O2—C35—C34	4.1 (4)
C25'—C24'—C29'—N6'	−178.8	C37—O2—C35—C36	−177.1 (3)
N4'—C24'—C29'—C28'	177.7	C34—C35—C36—C31	1.4 (5)
C25'—C24'—C29'—C28'	−1.2	O2—C35—C36—C31	−177.4 (3)
N5'—N6'—C30'—C31	−80.7 (7)	C32—C31—C36—C35	−1.3 (5)
C29'—N6'—C30'—C31	102.5 (7)	C30'—C31—C36—C35	178.1 (6)
C6—C1—N1—N2	−0.3 (5)	C30—C31—C36—C35	−176.5 (4)
C2—C1—N1—N2	179.0 (4)	C35—O2—C37—C19	174.0 (3)
C1—N1—N2—N3	−0.8 (5)	C18—C19—C37—O2	−73.4 (3)
N1—N2—N3—C6	1.6 (4)	C20—C19—C37—O2	107.0 (3)
N1—N2—N3—C7	177.2 (3)	N8—N9—C38—C39	−179.1 (3)
C43—N7—N8—N9	0.1 (4)	C44—N9—C38—C39	−3.9 (5)
N7—N8—N9—C38	0.1 (3)	N8—N9—C38—C43	−0.3 (3)
N7—N8—N9—C44	−175.7 (3)	C44—N9—C38—C43	175.0 (3)
N1—C1—C2—C3	178.0 (5)	N9—C38—C39—C40	178.5 (3)
C6—C1—C2—C3	−2.8 (7)	C43—C38—C39—C40	−0.2 (5)
C1—C2—C3—C4	1.8 (9)	C38—C39—C40—C41	−0.7 (5)
C2—C3—C4—C5	0.1 (10)	C39—C40—C41—C42	0.7 (6)
C3—C4—C5—C6	−1.2 (7)	C40—C41—C42—C43	0.3 (6)
N2—N3—C6—C1	−1.7 (4)	N8—N7—C43—C42	177.7 (4)
C7—N3—C6—C1	−176.8 (3)	N8—N7—C43—C38	−0.3 (4)
N2—N3—C6—C5	178.2 (4)	C41—C42—C43—N7	−178.9 (4)
C7—N3—C6—C5	3.1 (6)	C41—C42—C43—C38	−1.1 (5)
N1—C1—C6—N3	1.2 (4)	N9—C38—C43—N7	0.4 (3)
C2—C1—C6—N3	−178.0 (4)	C39—C38—C43—N7	179.3 (3)
N1—C1—C6—C5	−178.7 (3)	N9—C38—C43—C42	−177.9 (3)
C2—C1—C6—C5	2.0 (6)	C39—C38—C43—C42	1.1 (5)
C4—C5—C6—N3	−179.8 (4)	C38—N9—C44—C45	82.8 (4)
C4—C5—C6—C1	0.1 (5)	N8—N9—C44—C45	−102.3 (3)
C6—N3—C7—C8	80.2 (5)	N9—C44—C45—C50	−131.3 (3)
N2—N3—C7—C8	−94.4 (4)	N9—C44—C45—C46	53.2 (4)
N3—C7—C8—C9	−154.6 (3)	C50—C45—C46—C47	−0.5 (5)
N3—C7—C8—C13	27.8 (5)	C44—C45—C46—C47	174.9 (3)
C13—C8—C9—C10	−0.7 (5)	C45—C46—C47—C48	−0.8 (5)
C7—C8—C9—C10	−178.4 (3)	C46—C47—C48—C49	0.5 (5)
C14—O1—C10—C11	−0.8 (4)	C54—O3—C49—C48	−20.2 (4)
C14—O1—C10—C9	177.7 (3)	C54—O3—C49—C50	162.8 (3)
C8—C9—C10—C11	−0.2 (5)	C47—C48—C49—O3	−175.7 (3)
C8—C9—C10—O1	−178.7 (3)	C47—C48—C49—C50	1.1 (5)
O1—C10—C11—C12	179.6 (3)	C46—C45—C50—C49	2.1 (4)
C9—C10—C11—C12	1.2 (5)	C44—C45—C50—C49	−173.4 (3)
C10—C11—C12—C13	−1.3 (5)	O3—C49—C50—C45	174.7 (2)
C11—C12—C13—C8	0.4 (6)	C48—C49—C50—C45	−2.4 (4)
C9—C8—C13—C12	0.6 (5)	C49—O3—C54—C17	161.4 (3)
C7—C8—C13—C12	178.3 (3)	C16—C17—C54—O3	94.9 (3)
C10—O1—C14—C15	173.4 (3)	C18—C17—C54—O3	−86.4 (3)
O1—C14—C15—C20	−98.4 (3)		

### Hydrogen-bond geometry (Å, °)

**Table:**

*D*—H···*A*	*D*—H	H···*A*	*D*···*A*	*D*—H···*A*
C2—H2···N8^i^	0.93	2.48	3.376 (7)	163.
C27—H27···N1^ii^	0.93	2.54	3.453 (7)	166.

Symmetry codes: (i) *x*+1, *y*−1, *z*+1; (ii) *x*, *y*+1, *z*−1.
